# A human-specific VNTR in the *TRIB3* promoter causes gene expression variation between individuals

**DOI:** 10.1371/journal.pgen.1008981

**Published:** 2020-08-03

**Authors:** Tiit Örd, Tarmo Puurand, Daima Örd, Tarmo Annilo, Märt Möls, Maido Remm, Tõnis Örd

**Affiliations:** 1 Estonian Biocentre, Institute of Genomics, University of Tartu, Tartu, Estonia; 2 Institute of Molecular and Cell Biology, University of Tartu, Tartu, Estonia; 3 Estonian Genome Center, Institute of Genomics, University of Tartu, Tartu, Estonia; 4 Institute of Mathematics and Statistics, University of Tartu, Tartu, Estonia; University of Melbourne, AUSTRALIA

## Abstract

Tribbles homolog 3 (TRIB3) is pseudokinase involved in intracellular regulatory processes and has been implicated in several diseases. In this article, we report that human *TRIB3* promoter contains a 33-bp variable number tandem repeat (VNTR) and characterize the heterogeneity and function of this genetic element. Analysis of human populations around the world uncovered the existence of alleles ranging from 1 to 5 copies of the repeat, with 2-, 3- and 5-copy alleles being the most common but displaying considerable geographical differences in frequency. The repeated sequence overlaps a C/EBP-ATF transcriptional regulatory element and is highly conserved, but not repeated, in various mammalian species, including great apes. The repeat is however evident in Neanderthal and Denisovan genomes. Reporter plasmid experiments in human cell culture reveal that an increased copy number of the *TRIB3* promoter 33-bp repeat results in increased transcriptional activity. In line with this, analysis of whole genome sequencing and RNA-Seq data from human cohorts demonstrates that the copy number of *TRIB3* promoter 33-bp repeats is positively correlated with *TRIB3* mRNA expression level in many tissues throughout the body. Moreover, the copy number of the *TRIB3* 33-bp repeat appears to be linked to known *TRIB3* eQTL SNPs as well as *TRIB3* SNPs reported in genetic association studies. Taken together, the results indicate that the promoter 33-bp VNTR constitutes a causal variant for *TRIB3* expression variation between individuals and could underlie the results of SNP-based genetic studies.

## Author summary

The current article is devoted to a previously undescribed genetic element residing in the promoter region of human *TRIB3* gene and consisting of a variable number of tandemly repeated 33-bp segments. We found that human genome contains from 1 to 5 copies of the repeat and that the copy number distribution varies in human populations around the world. Importantly, an increased number of copies associated with increased expression of the *TRIB3* gene in many tissues of the body, giving rise to variation in *TRIB3* gene expression between individuals. Mechanistically, the 33-bp segment possesses the ability to activate transcription, and it becomes more potent as it is repeated. The 33-bp segment is repeated in Neanderthals and Denisovans, but not in great apes or other mammals, suggesting that the expansion of the 33-bp unit in the *TRIB3* promoter occurred after the split of the hominin and chimpanzee lineages. The TRIB3 protein participates in multiple biological processes as a regulatory factor and is linked to several diseases. Thus, the importance of our study is to shed light on a new mechanism determining *TRIB3* expression level variation between individuals, and, more generally, to highlight how tandem repeat variations can serve as regulatory variants.

## Introduction

The pseudokinase Tribbles homolog 3 (TRIB3) is known to interact and modulate the function of several transcription factors, protein kinases, ubiquitin ligases and other proteins (for review, see [1]). Through these interactions, TRIB3 has been reported to participate in processes such as the regulation of cellular stress response and cell death [2–5], megakaryocytopoiesis [6], metabolic adaptation to nutrient-limiting conditions [7], and to mediate cancer cell sensitivity to chemotherapeutics [8–13]. Moreover, TRIB3 has been implicated in the pathogenesis of several diseases, including type 2 diabetes and its complications [14–18], Parkinson’s disease [19, 20] and several types of cancer [21–23]. Recently, two novel associations relating *TRIB3* genetic variants to disease have been described. Lorenzi et al. [24] identified a link between *TRIB3* and the stereotypical pattern of gray matter loss in Alzheimer’s disease, and Yamada et al. [25] identified *TRIB3* as a susceptibility locus for ischemic stroke in an exome-wide association study. Therefore, there is a considerable interest to improve our understanding of mechanisms which govern human *TRIB3* expression and its variation between individuals.

Variable number tandem repeats (VNTRs) are defined as continuously located repeated DNA sequence motifs with the unit size ≥7 bp. VNTRs evolve as a result of recombination or replication slippage events and there are about ten thousand polymorphic VNTRs in the human genome [26]. It has been found that the unit copy number polymorphism of these loci can have a significant impact on the properties of genes, including gene expression level [27, 28]. Due to the repetitive nature of VNTRs, the analysis of their structure by short-read high-throughput sequencing is complicated [29]. In the current article, we report that the human *TRIB3* promoter contains a 33-bp VNTR and characterize this genetic element using a k-mer counting approach to analyze short-read whole genome sequencing (WGS) data as well as search for sequencing reads spanning the VNTR region for direct allele detection. As a result of the analyses, we uncover that alleles with a higher number of the 33-bp repeat copies generate higher levels of *TRIB3* expression and that variation in the copy number of the 33-bp repeat could represent the causal variant for a number of single nucleotide polymorphisms (SNP-s) that have been linked to variation in *TRIB3* expression level in various tissues of the body.

## Results

### A VNTR with repeat length of 33 bp resides in the promoter of the human *TRIB3* gene

**Fig 1:**
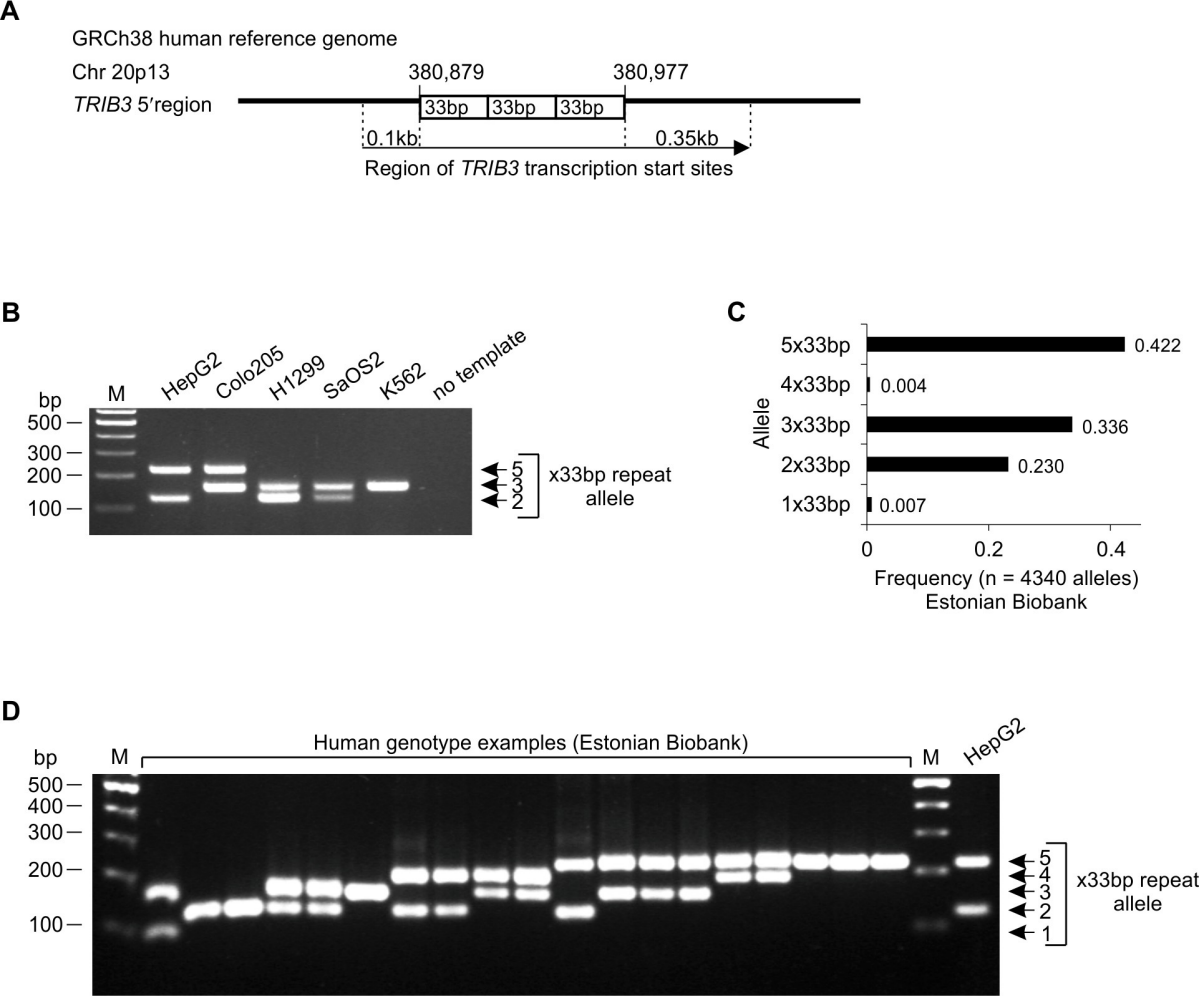
Characterization of the allelic heterogeneity of the 33-bp VNTR in the human *TRIB3* promoter. **(A)** A schematic of the *TRIB3* gene 5’ region showing the location of VNTR, containing 3 copies of the 33-bp repeat as in the human reference genome (GRCh38). **(B)** PCR analysis of the *TRIB3* promoter 33-bp repeat copy number in human cell lines. The DNA of hepatocellular carcinoma (HepG2), colorectal adenocarcinoma (Colo205), non-small cell lung carcinoma (H1299), osteosarcoma (SaOS2) and chronic myelogenous leukemia (K562) was amplified using primers flanking the VNTR and the products were separated by agarose gel electrophoresis. **(C)** Human *TRIB3* promoter 33-bp VNTR allele frequencies in Estonian Biobank participants, based on whole genome sequencing data. **(D)** PCR analysis of the 33-bp repeat copy number in a selection of Estonian Biobank participants. The DNA was analyzed as in panel B, and HepG2 is shown as a control. Lane M in panels B and D contains a molecular weight marker (100 bp DNA ladder).

During previous studies of human *TRIB3* gene, we observed that the 5’ region of the gene contains a variable number of tandemly repeated segments with the length of 33 bp [30]. *TRIB3* transcripts are initiated at several sites extending from about 0.1 kb upstream to 0.35 kb downstream of the 33-bp repeats [30, 31]; thus, the repeats overlap with the promoter region of the gene (Fig 1A).

The GRCh38 human reference genome contains 3 copies of this repeating element, spanning positions 380,879 to 380,977 of chromosome 20. To assess the copy number heterogeneity of this repeat, a PCR amplicon using primers flanking the VNTR was designed. Analysis of genomic DNA from commonly used human cell lines reveals several examples of heterozygosity at the 33-bp repeat, with alleles of 2 and 5 copies present in HepG2, 3 and 5 copies in Colo205, and 2 and 3 copies in H1299 and SaOS2; in the case of K562 cells, only the 3-copy allele is detectable (Fig 1B).

To investigate the copy number variation of the *TRIB3* 33-bp repeats in a general population, we utilized sequencing data and DNA samples from the biobank of the Estonian Genome Center of University of Tartu (EGCUT) [32]. A 150-bp read length WGS data set comprised of >2000 individuals from the Estonian population was used to estimate allele frequencies [33]. From the 150 bp reads, alleles up to 4x33bp were called by searching for spanning reads; since alleles with ≥5 copies of the 33-bp repeat cannot be fully spanned with 150 bp read length, partial spanning was accepted and any reads supporting >4x33bp were designated 5x33bp. The results reveal that in the Estonian population, alleles with 2, 3 or 5 copies of the repeating element all appear to be quite frequent, with 5x33bp being the most common (allele frequency 0.42), followed by 3x33bp (0.34) and 2x33bp (0.23) (Fig 1C). This analysis further uncovered that alleles with 1 or 4 copies of the repeating element also occur, albeit at an allele frequency of less than 1% (Fig 1C).

A selection of individuals carrying the rare (1 or 4 repeats) alleles of the 33-bp repeat based on the WGS were subjected to follow-up PCR analysis, along with examples of individuals carrying the common alleles (2, 3 or 5 repeats). The results of PCR product gel electrophoresis confirmed the occurrence of the 1x33bp and 4x33bp alleles in these individuals (Fig 1D). We additionally sought to uncover potential alleles with >5 copies of the 33-bp repeat by using a k-mer counting technique (described below) on the EGCUT WGS data set; however, PCR-based follow-up of 11 highest ranking individuals (33-bp copy number estimates ranging from 6.13 to 6.69) revealed all of them to be 5x33bp homozygotes (S1 Fig). Similarly, searching for sequencing reads containing a full coverage of four 33-bp repeats and, at either end of that, partial matches for additional 33-bp copies (indicative of a hypothetical ≥6x33bp allele) did not uncover any individuals from the EGCUT WGS data set.

### Evolutionary aspects of the human *TRIB3* promoter 33-bp repeats: the highly conserved 33-bp segment is repeated also in Neanderthal and Denisovan hominins, but exists in a single copy in other mammalian genomes

**Fig 2:**
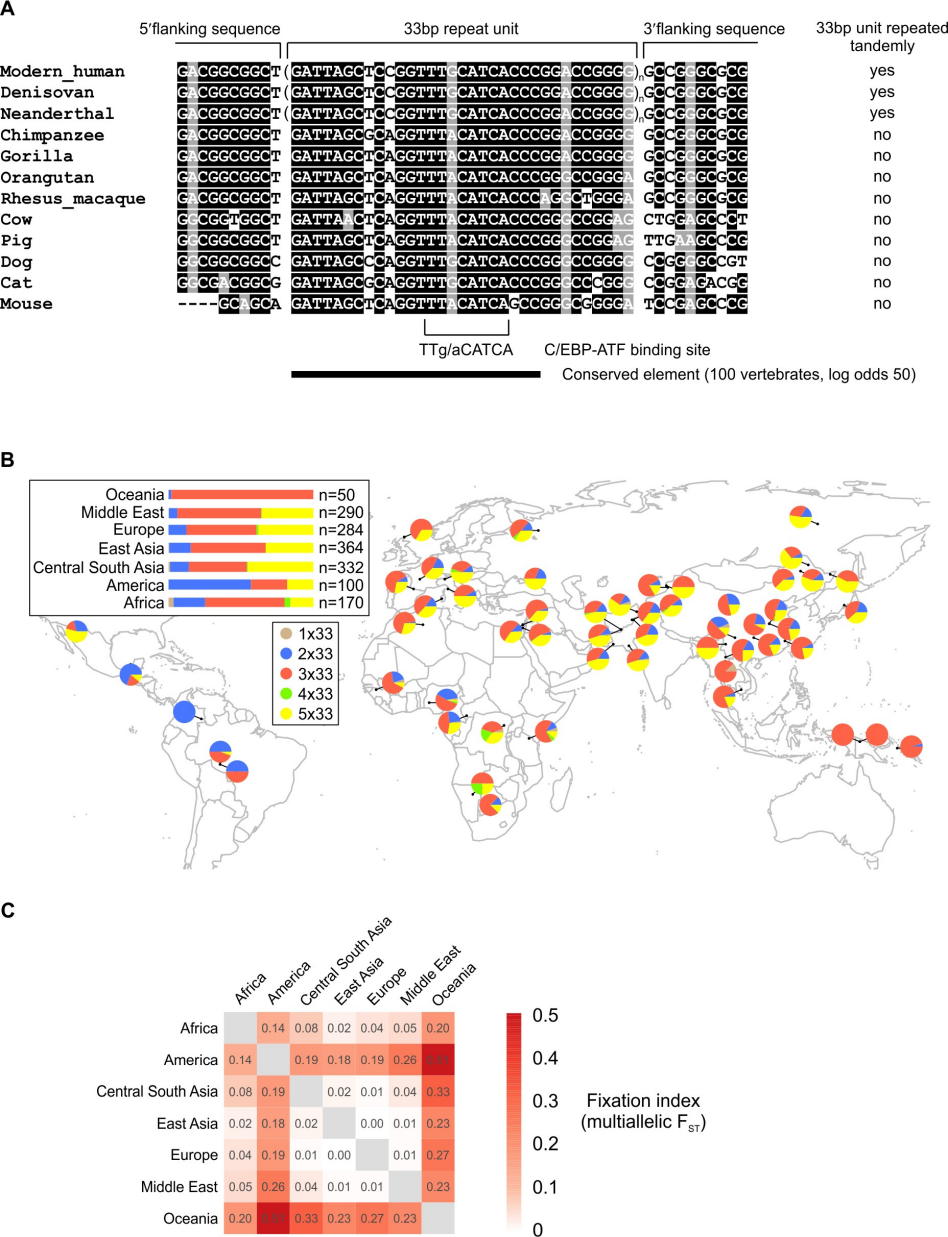
Evolutionary conservation of the *TRIB3* promoter 33-bp VNTR area across mammalian species and the geographical distribution of the *TRIB3* 33-bp VNTR alleles in human populations. **(A)** Nucleotide sequence alignment of the human *TRIB3* promoter 33-bp VNTR repeat unit and flanking areas with the corresponding sequence in various mammalian genomes. Only one copy of the repeat is shown for modern human and Denisovan and Neanderthal hominins. Alignment was produced using Clustal Omega and Boxshade, with identical nucleotides shown in black and conserved purines in grey. The location of the C/EBP-ATF binding site is highlighted, overlapping a highly conserved sequence element. **(B)** A map showing the allele frequency distribution of the human *TRIB3* promoter 33-bp VNTR repeat in various populations around the world. The results are based on data from the Human Genome Diversity Project (HGDP). **(C)** Multi-allelic fixation index (*F*_ST_) for *TRIB3* 33-bp repeat copy number alleles in HGDP superpopulations.

The inspection of mammalian genomes reveals that a segment identical or very similar to the 33-bp repeat element of human *TRIB3* promoter is present in the 5’ region of *TRIB3* genes from species representing diverse mammalian orders, such as primates, artiodactyls (cow and pig), carnivorans (dog and cat) and rodents (mouse) (Fig 2A). In particular, sequence conservation is strong in the first half of the 33bp segment, which is noted as a constrained element with a log odds score of 50 by PHAST analysis of 100 vertebrate genomes [34, 35].

Remarkably, the high-coverage genomes of Neanderthal and Denisovan hominins [36, 37] reveal that the *TRIB3* 5’ regions of these extinct archaic humans contain an allele with at least three copies of the 33-bp repeat, while the *TRIB3* 5’ regions of the great apes chimpanzee, gorilla and orangutan contain only single copy of this 33-bp segment, similarly to the other mammalian orders (Fig 2A). Thus, the data suggests that the tandem repeat expansion of the 33-bp segment seen in the human *TRIB3* promoter occurred after the divergence of the hominins and the great apes but before the divergence of the three branches of hominins. The scarcity of Neanderthal and Denisovan genomic data does not allow a more detailed analysis of these populations. Interestingly, the currently available high-coverage Denisovan genome contains a single nucleotide variation (SNV) in position 10 of the third copy of the 33-bp repeats (heterozygous, C/A). A matching SNV has been reported in modern humans as well, however it is a highly rare variant (rs867414698; gnomAD study-wide alternative allele frequency 0.0021 [38]).

As the genetic variation in modern humans tends to cluster with geographic origin, we also assessed the WGS data generated by Human Genome Diversity Project (HGDP) [39] for *TRIB3* 33-bp repeat alleles, using the approach described above for EGCUT data. The results, based on approx. 800 individuals spanning 54 geographically distinct world populations, reveal considerable variation in allele frequencies (Fig 2B). In many Asian, European and Middle Eastern populations, the 3x33bp and 5x33bp alleles are both quite frequent and can rank as the most common allele in a population (Fig 2B). The 2x33bp allele appears at a particularly high frequency in some American populations, while the Oceanic populations show nearly exclusively the 3x33bp allele (Fig 2B). For the 1x33bp and 4x33bp alleles, African populations display a noticeably higher incidence compared to populations from other continents (Fig 2B). To evaluate how much of the copy number variation in the *TRIB3* 33-bp repeat can be explained by population structure, we calculated the multi-allelic fixation index *F*_ST_, as defined in [40], for pairwise comparisons between the seven superpopulations in HGDP. The results show that there is considerable population differentiation, particularly in Oceania and America relative to the other superpopulations (Fig 2C).

### *TRIB3* promoter variants containing more copies of the 33-bp repeat show increased transcriptional activity

The conservation of the *TRIB3* promoter 33-bp repeat sequence throughout diverse mammalian orders suggests functional importance. In the center of the 33-bp segment, at positions 14 to 21, resides a motif TT(G/A)CATCA, which corresponds to a transcriptional regulatory motif termed the C/EBP-ATF response element (CARE) [30] (Fig 2A). CARE-s mediate gene activation in response to cellular stresses such as endoplasmic reticulum stress, oxidative stress, nutrient deficiency and viral infection [41, 42]. A key transcription factor for driving CARE-dependent gene expression is considered to be Activating Transcription Factor 4 (ATF4), acting as a heterodimer with various C/EBP factors [43].

**Fig 3:**
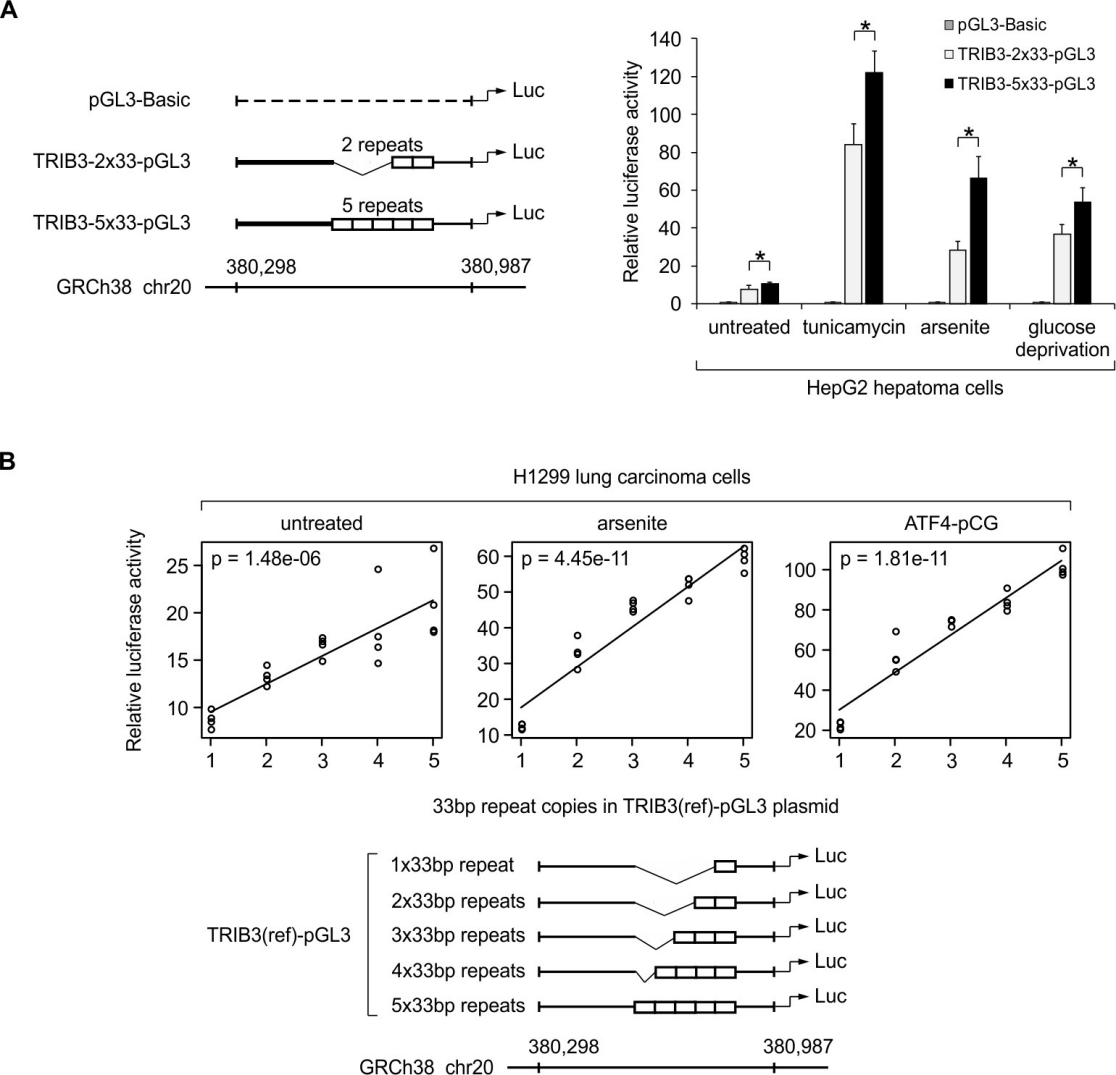
Human *TRIB3* promoter variants containing more copies of the 33-bp repeat demonstrate elevated transcriptional activity in luciferase reporter assays. **(A)** Results from HepG2 cells transfected with human *TRIB3* promoter reporter constructs containing two (TRIB3-2x33-pGL3) or five (TRIB3-5x33-pGL3) copies of the 33-bp repeat. Cells were either left untreated or treated with tunicamycin (2 μg/ml, 10 h) or arsenite (20 μM, 10 h), or subjected to glucose deprivation (10 h). Luciferase activities are shown as mean ± SD from 4 independent transfections. *P < 0.05 comparing allelic variants using two-tailed *t* test followed by Holm–Bonferroni correction. **(B)** Reporter constructs carrying 1 to 5 copies of the 33-bp repeat were transfected into H1299 cells and their transcriptional activity was assayed under control condition (untreated), oxidative stress (30 μM arsenite, 10 h), and with co-transfection of an ATF4 overexpression plasmid (ATF4-pCG). Four independent transfections were performed, and the relationship between 33-bp copy number and luciferase activity was modeled using linear regression. For both panels, luciferase activities are presented relative to the activity of empty reporter vector (pGL3-Basic without *TRIB3* fragment) under the same conditions. The genomic coordinates (GRCh38 human reference genome) of the region studied in the reporter assay are indicated below the plasmids.

To study whether the copy number of 33-bp repeats affects *TRIB3* promoter activity, we generated luciferase reporter constructs which differ by the number of 33-bp repeats. The constructs contain human *TRIB3* gene segments up- and downstream of the 33-bp repeats (580 bp and 10 bp, respectively). For Fig 3A, the plasmids contain either two copies (plasmid TRIB3-2x33-pGL3) or five copies of the 33-bp repeat (TRIB3-5x33-pGL3), and both plasmids differ from the GRCh38 reference genome by six common SNPs that are listed in the Materials and Methods. The plasmids were transfected into HepG2 human hepatoma cells and the cells were either left untreated or treated with tunicamycin, an inducer of endoplasmic reticulum stress, with arsenite, an inducer of oxidative stress, or with glucose-free growth medium, to induce nutrient deficiency.

As shown in Fig 3A, the plasmids containing the *TRIB3* promoter fragments are strongly stress-responsive, upregulating the expression of reporter gene in all three treatments. The largest increase is observed in tunicamycin-treated cells, while arsenite exposure and glucose starvation result in slightly lower activation of reporter (Fig 3A). Importantly, in the cells transfected with plasmid TRIB3-5x33bp-pGL3 and exposed to stressors (tunicamycin, arsenite or glucose-free medium), the reporter activity is significantly higher than in the cells transfected with TRIB3-2x33-pGL3 and exposed to the same treatments (Fig 3A). Additionally, the 5-repeat allele also demonstrates increased luciferase activity compared to the 2-repeat allele in unstressed cells (Fig 3A).

To compare the full series of discovered copy number variants of the 33-bp repeat, a second set of luciferase reporter plasmids was prepared (Fig 3B), containing 1–5 copies of the *TRIB3* 33-bp repeat surrounded by flanking sequences that are identical to the GRCh38 reference genome (e.g., regarding SNPs in the region). To broaden the scope of the study, these plasmids were tested using the H1299 human non-small cell lung carcinoma cell line and the overexpression of ATF4 was included as a treatment. As depicted in Fig 3B, in the control cells as well as in the cells subjected to arsenite stress or overexpression of ATF4, the strength of the luciferase reporter signal positively and significantly correlates with the number of 33-bp repeats in the transfected reporter plasmid. Notably, increasing the 33-bp copies from 1 to 5 leads to approximately 5-fold increase in transcriptional activity under conditions that stimulate CARE activity (arsenite and ATF4 overexpression), indicating that transcriptional output increases quite steeply as a function of the 33-bp copy number. Thus, the results demonstrate that increased copy number of the 33-bp repeat results in elevated *TRIB3* promoter activity.

### Evidence for an *in-cis* regulatory effect of the *TRIB3* 33-bp repeat towards *TRIB3* gene expression level from paired RNA-Seq and WGS data: k-mer-based repeat copy number estimation

To study the effect of *TRIB3* 33-bp repeat copy number variation on the gene expression level in the human body, we turned to data sets with RNA-Seq and WGS data from the same individuals. To effectively use WGS data with read lengths <150 bp (e.g., 100 bp, in which case a read cannot reliably span even 3x33 bp), we switched to a k-mer counting based approach for quantifying the *TRIB3* 33-bp repeat copy number. The k-mer method estimates the average *TRIB3* 33-bp repeat copy number across each individual’s two alleles by querying the WGS reads for the occurrence of 25-mer oligonucleotides derived from the *TRIB3* 33-bp segment sequence, normalizing the counts to those of 25-mers derived from sequences neighboring the 33-bp repeat region.

**Fig 4:**
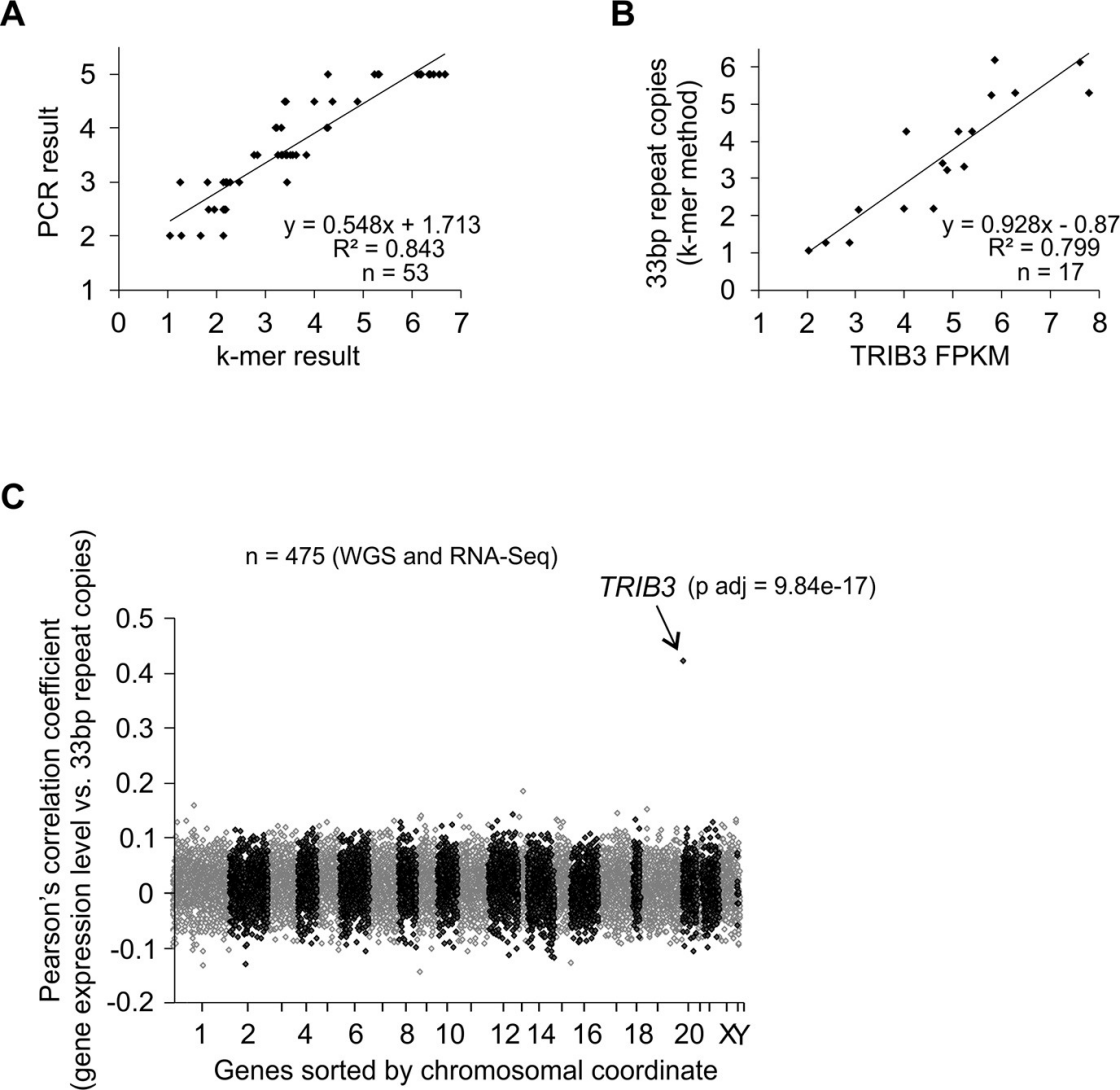
*TRIB3* 33-bp repeat copy number is positively correlated with the expression level of *TRIB3* in genome-wide gene expression profiling. **(A)** Validation of a k-mer counting-based method for the estimation of *TRIB3* 33-bp repeat copy number from whole genome sequencing data. The k-mer based copy number estimates are compared to the results of PCR product gel analysis from the same individuals. The PCR results are shown as the average repeat copy number from the diploid genotype of each individual. **(B)** Correlation of *TRIB3* 33-bp copy number to *TRIB3* gene expression in the subset of individuals with PCR-validated 33-bp repeat genotypes. Individuals from panel A that further had blood RNA-Seq data available are included. **(C)** Across all genes, the copy number of the *TRIB3* 33-bp repeat is correlated specifically with the expression level of *TRIB3* mRNA. Correlation between gene expression and the copy number of the *TRIB3* 33-bp repeat was analyzed for all genes expressed in blood, as determined by RNA-Seq (total 13896 genes). For the panels of this figure, the genotyping samples and the DNA and RNA sequencing data originate from the Estonian Biobank.

We first analyzed individuals from the EGCUT cohort, where blood RNA-Seq has been performed for a subset of individuals analyzed by WGS [32, 44]. Analysis using PCR-validated individuals reveals a good agreement between the 33-bp repeat copy number values obtained from PCR and WGS k-mer counting across the range of possible values (Fig 4A). Further, for the PCR-validated individuals with RNA-Seq available, *TRIB3* mRNA expression level from blood reveals a positive correlation to k-mer based 33bp copy number estimate (Fig 4B). Therefore, we extended this analysis to n = 475 blood samples and all expressed genes as detected by RNA-Seq. The results depicted in Fig 4C reveal that out of all expressed genes, the copy number of the *TRIB3* 33-bp repeat shows the strongest correlation with the expression level of *TRIB3* itself, yielding a significant and positive correlation (Pearson's r = 0.42, corrected p = 9.8e-17), with no other genes demonstrating a correlation of |r|>0.2.

### The copy number of *TRIB3* promoter 33-bp repeats positively correlates with *TRIB3* mRNA expression level in many tissues

**Fig 5:**
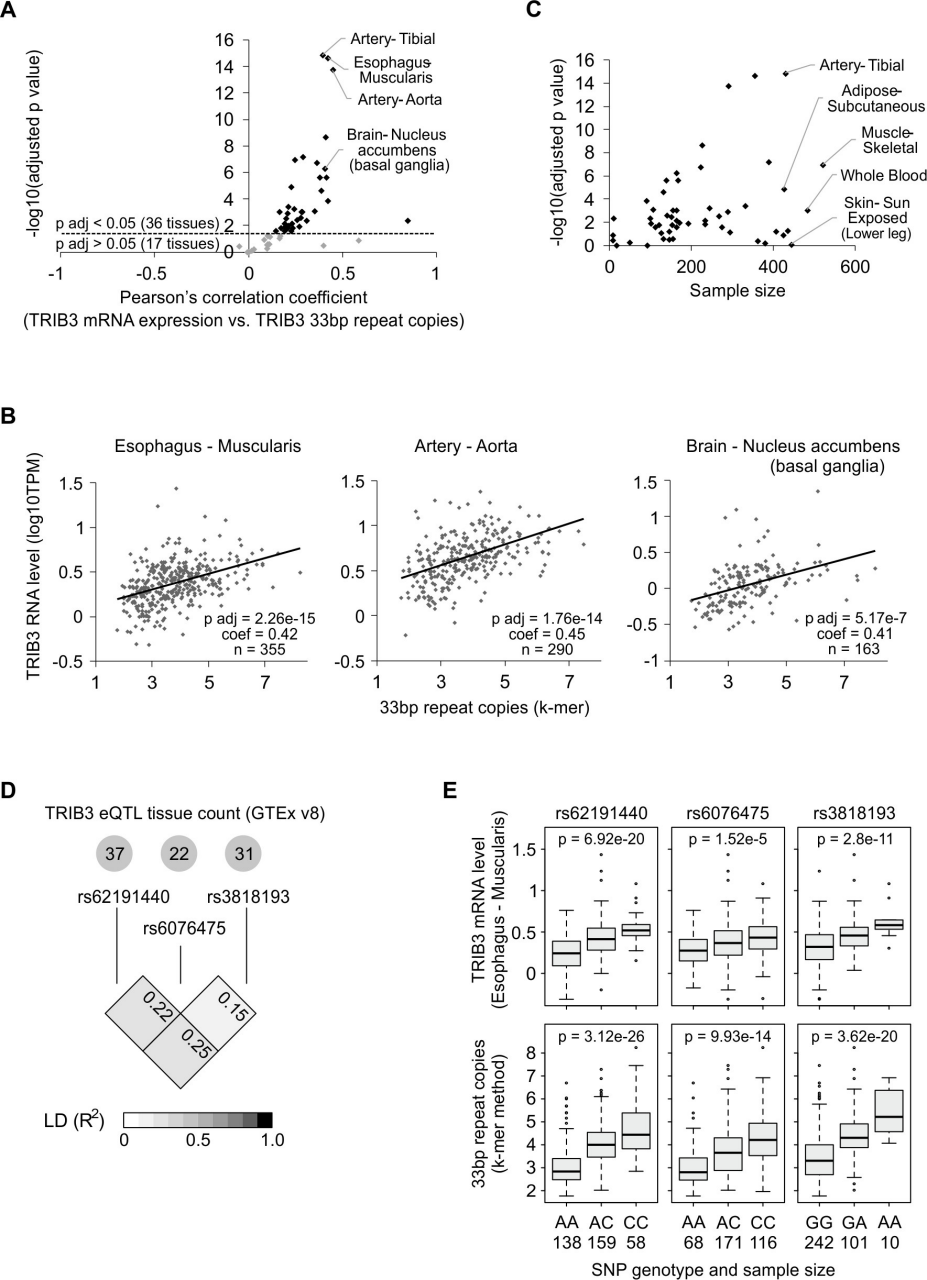
Variation in the *TRIB3* 33-bp repeat copy number is linked to *TRIB3* expression level in various tissues of the body. **(A)** Correlation between *TRIB3* 33-bp repeat copy number and *TRIB3* gene expression level in 53 tissues from the Genotype-Tissue Expression (GTEx) v8 data set. The analysis included all tissues with ≥5 donors with RNA-Seq and whole genome sequencing from the same donor. The k-mer counting method was used to estimate the *TRIB3* 33-bp repeat copy number from genome sequencing reads. False discovery rate was used to correct the p values. **(B)** Scatter plots from selected tissues from panel A, showing the correlation between *TRIB3* 33-bp repeat copy number and *TRIB3* mRNA expression at the level of individuals donors. **(C)** The strength of the association between 33-bp repeat copy number and *TRIB3* expression level in different tissues (the correlation p value) does not appear to be driven strictly by the available sample size. All tissues from panel A are shown, and a selection of tissues with >400 samples are labeled. **(D)** Linkage disequilibrium results for SNPs that are *TRIB3* eQTLs in multiple tissues. The eQTL tissue counts were obtained from the GTEx v8 data release. LD was calculated from GTEx donor genotypes. **(E)** Known SNP eQTLs for *TRIB3* expression level demonstrate associations with the *TRIB3* 33-bp repeat copy number. Statistical significance was calculated by linear regression for the SNP allele effect.

To assess whether the correlation between *TRIB3* 33-bp repeat copy number and *TRIB3* mRNA level also occurs in other human tissues besides blood, we used the Genotype-Tissue Expression (GTEx) v8 data set [45]. All tissues with ≥5 samples with RNA-Seq and WGS were included, yielding 53 different tissues for analysis. Out of these, a total of 36 tissues revealed a significant (p adjusted < 0.05) correlation between genomic *TRIB3* 33-bp repeat copy number and *TRIB3* mRNA expression in that tissue (Fig 5A, S1 Table), indicating a broad tissue distribution for the effect. Notably, in all tissues where a significant correlation was found, the direction of the correlation was positive (Fig 5, S1 Table). Tissues that yielded the most highly statistically significant results include arterial tissues and esophagus muscularis, characterized by high smooth muscle content; however, other tissues not rich in smooth muscle also ranked highly, such as nucleus accumbens of the brain (Fig 5B). The number of RNA-Seq samples available for each tissue varied greatly; however, some tissues with considerably large sample sizes still show a lack of association, implicating mechanisms other than the 33-bp repeat copy number as major determinants of *TRIB3* expression level in those tissues (Fig 5C).

The GTEx project has provided a wealth of SNP expression quantitative trait loci (eQTL) data for *TRIB3* expression. We filtered the GTEx v8 eQTL SNPs for *TRIB3* expression for SNPs that would be eQTLs in multiple tissues (≥20) and that would be largely independent of each other in terms of genetic linkage. This resulted in a set of 3 SNPs, rs62191440, rs6076475 and rs3818193, which are GTEx v8 eQTLs for *TRIB3* in 37, 22 and 31 tissues, respectively, and have LD values of R^2^ < 0.3 between each other (Fig 5D). To assess whether the effects of these SNPs might be linked to variations in *TRIB3* 33-bp repeat copy number, we selected all GTEx v8 individuals that had SNP genotypes, WGS and esophagus muscularis RNA-Seq available for analysis. The results depicted in Fig 5E reveal that for these *TRIB3* eQTL SNPs, individuals carrying the SNP allele associated with increased *TRIB3* mRNA expression tend to also have a higher estimated *TRIB3* 33-bp repeat copy number, indicating that variation in the *TRIB3* promoter VNTR could underlie the eQTL associations that have been discovered for these SNPs.

**Fig 6:**
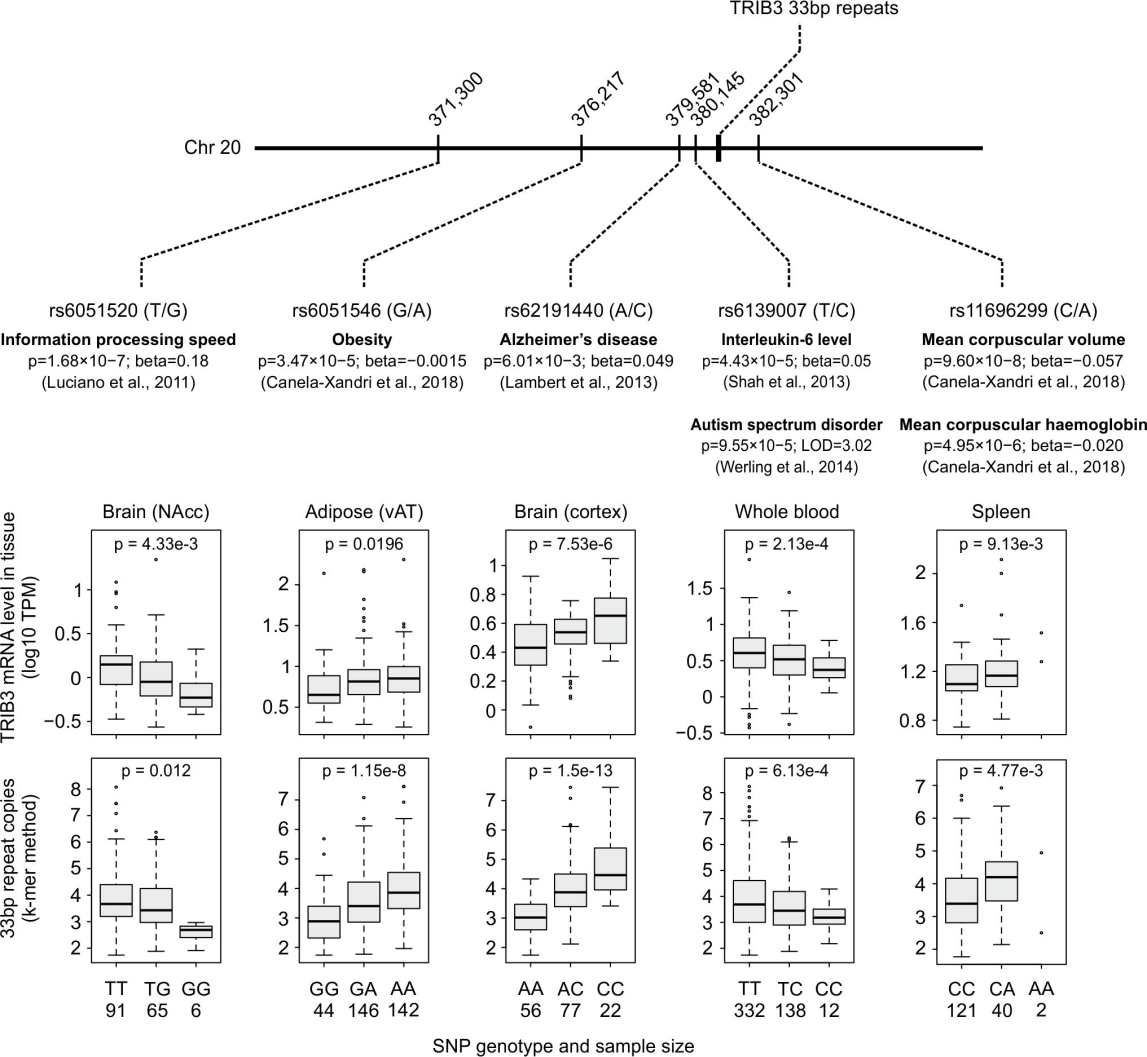
*TRIB3* SNPs highlighted by genetic association studies are linked to 33-bp repeat copy number as well as *TRIB3* mRNA expression in phenotype-relevant tissues. SNPs localizing near the *TRIB3* 33-bp repeat and associated (or suggestive) for human phenotypes based on GWAS, PheWAS or other genetic association studies are shown. Using RNA-Seq and whole genome sequencing data from GTEx v8, the association between the lead SNP genotype and *TRIB3* RNA expression level or *TRIB3* 33-bp repeat copy number was calculated using linear regression for the SNP allele effect. The k-mer counting method was used to estimate the *TRIB3* 33-bp repeat copy number from genome sequencing reads.

We searched literature for SNPs that are located in the vicinity of the *TRIB3* 33-bp repeat region and that have been noted as significant or suggestive in genetic association studies, including GWAS [46], UK Biobank PheWAS [47], linkage [48] and other types of genetic association studies [47, 49–51]. This uncovered 5 SNPs that have been associated with phenotypes, including several blood and nervous system traits or diseases, in genome-wide or more tightly focused studies (Fig 6). Analysis of the 5 association study lead SNPs in GTEx WGS data reveals positive as well as negative associations between the SNP alternative allele and the copy number of the *TRIB3* 33-bp repeat (Fig 6), indicating that the lead SNPs could be tagging various alleles of the 33-bp repeat. Further, GTEx RNA-Seq data from phenotype-relevant tissues reveals that these SNPs are linked to *TRIB3* gene expression with directionality that is in line with their association to *TRIB3* 33-bp copy number (Fig 6), indicating that 33-bp repeat alleles could underlie the effects of these SNPs on *TRIB3* expression level in tissues relevant to the reported trait or disease.

## Discussion

In the current era of human genetic studies, powered mainly by short-read sequencing and SNP microarrays, VNTRs have been addressed comparatively less than other types of variants due to technological constraints. Nevertheless, more than a hundred thousand polymorphic tandem repeats have been described that localize within or near genes in the human genome, representing considerable potential to alter the protein coding or regulatory sequences of genes [52]. In the current article, we characterize copy number variation in a 33-bp tandem repeat localized to the promoter region of human *TRIB3* and uncover that alleles with a higher copy number of the 33-bp repeating element lead to elevated *TRIB3* gene expression level.

The human reference genome (GRCh38) contains 3 identical tandemly repeating elements of the *TRIB3* 33-bp VNTR. Alleles with 2 or 5 copies are also relatively frequent in many human populations. Crucially, this 33-bp region that has been repeated in humans contains a highly conserved sequence element, which likely accounts for the effect of 33-bp VNTR copy number variation on *TRIB3* mRNA expression level. Previous functional studies of the human *TRIB3* (as well as mouse *Trib3*) promoter sequences have identified that a C/EBP-ATF composite motif, which rests within the highly conserved part of the 33-bp repeat, represents a crucial transcription factor binding site for driving *TRIB3* mRNA expression [30, 53]. A key factor shown to bind to this site is ATF4 [5, 30, 53], a bZIP transcription factor with several known roles in physiology and disease [54–56]. Thus, *TRIB3* alleles with more copies of the 33-bp repeat can convey increased expression of *TRIB3* by having more copies of the C/EBP-ATF binding site, a positive regulatory element, in their promoter. In support of this, we find that the overexpression of ATF4 is sufficient to induce *TRIB3* promoter reporter constructs, and that the responsiveness to ATF4 (fold induction) increases steadily as the number of 33-bp repeats in the construct is increased step-wise from 1 to 5 copies, the range of natural variation.

Our finding that the great apes do not contain repeated copies of the 33-bp segment in the *TRIB3* promoter, while the archaic hominin branches Neanderthal and Denisovan do, allows us to estimate the time window when the VNTR evolved. The ancestors of hominins and chimpanzees split about 6 million years ago [57], and present-day humans diverged from Neanderthal and Denisovan lineages about 800,000 years ago [36, 58]. Thus, the expansion of the 33-bp segment in *TRIB3* promoter occurred within approximately 5 million years separating these two branching points of human evolution. All five of the copy number variants which we found in the current study are already present in the HGDP populations living in Africa, the ancestral home continent of modern humans, and the alleles 1x and 4x, the incidence of which is very low in most HGDP populations, are noticeably more frequent in Sub-Saharan Africa. Thus, the results suggest that all allelic variants ranging from 2x to 5x evolved before the migration of modern humans out of Africa, which, according to the earliest fossil evidence currently found, started about 200,000 years ago [59, 60].

Studies using knockout and overexpression models of mouse *Trib3* have led to TRIB3 being implicated in several biological processes *in vivo* (reviewed in [1, 61]). Promisingly, some of these results line up with significant or suggestive phenotypes from human genetic association studies. For example, enforced overexpression of *Trib3* in mouse adipose tissue has been reported to inhibit weight gain [62], and the human SNP rs6051546 alternative allele is associated with increased 33-bp repeat copy number, elevated *TRIB3* mRNA level in adipose tissue, and suggestively (p = 3.47e-5) [47] with reduced obesity. Several mouse and cellular models have implicated increased TRIB3 in neurodegeneration and neuronal cell death [19, 63, 64], and in humans, SNP associations with information processing speed (p = 1.68e-7; rs6051520) [50] and Alzheimer’s disease (p = 6.01e-3; rs62191440) [49] correlate with the expected effects of *TRIB3* 33-bp repeat copy number on *TRIB3* gene expression level in brain tissue. The SNP rs62191440 has received attention as a proxy for rs4813620 (R^2^ = 0.656 in 1000G EUR), which was reported by [24] to be associated with *TRIB3* expression in human brain tissues, but [65] found that Alzheimer’s disease status may affect this association. Human genetic association studies also link *TRIB3* to red blood cell parameters (p = 9.6e-8; rs11696299) [47], and we also uncovered a link to the 33-bp repeat copy count for this lead SNP. It has recently been reported that in mouse *Trib3* plays a role in red blood cell production; however, the effect appears to be complex, with alternate consequences under steady-state and myeloablative challenge conditions [66].

The results of our study allow to propose that a tandemly repeated polymorphic 33-bp repeat element in the human *TRIB3* gene gives rise to variation in *TRIB3* expression levels between individuals by multiplying the number of C/EBP-ATF binding sites in the *TRIB3* promoter. Further, this variation in the *TRIB3* 33-bp VNTR copy number appears to explain a number of SNP eQTLs for *TRIB3* expression in various tissues and could possibly also underlie *TRIB3* SNP effects from genetic association studies. Since the *TRIB3* 33-bp VNTR copy number is imperfectly associated with SNPs, direct genotyping of the alleles of the VNTR in large-scale study groups could allow for the discovery of stronger associations to human phenotypes.

## Materials and methods

### Ethics approval and consent to participate

The study was conducted in accordance with good ethical standards and was approved by the Research Ethics Committee of the University of Tartu (protocol number 289/T-10).

We used GTEx v8 (dbGap Accession phs000424.v8.p2) (Consent group: General Research Use) for the analyses described in this manuscript. The GTEx v8 DNA data were obtained from the GTEx Portal on September 27, 2019 and October 1–2, 2019, and the GTEx v8 RNA-seq data were obtained from the GTEx Portal on September 30, 2019.

### Analysis of human *TRIB3* promoter 33-bp repeat copy number by PCR

To study the human *TRIB3* promoter 33-bp repeat copy number in the genomic DNA of human cell lines and EGCUT biobank human DNA samples, DNA was amplified by PCR using primers 5’-CCACTTCCGCTGCGAGTCTCGTG-3’ and 5’-CCCGAGGGCATCAGACGGCG-3’, which bind to the regions flanking the repeats. In addition to the repeats, the PCR products contain fragments up- and downstream of the repeats (23 bp and 33 bp, respectively). The PCR products were analyzed by electrophoresis in 2.5% agarose gel and, in several cases, Sanger sequencing was also carried out, which confirmed that the products contain the human TRIB3 33-bp repeats.

### Human *TRIB3* 33-bp repeat copy number analyses from whole genome sequencing reads

Whole genome sequencing data was obtained from the Estonian Biobank [33], the Human Genome Diversity Project [39], and the GTEx project (v8 release) [45].

A k-mer counting approach was used to estimate the diploid allelic average copy number of the *TRIB3* 33-bp repeat from WGS reads. A list of 25-mer oligonucleotides was created from the genomic sequence of the *TRIB3* 33-bp repeat and its 5’ and 3’ flanking sequences using the glistmaker program of the GenomeTester4 package [67] with step of 1 bp, and k-mer frequencies were extracted from the list with the program glistquery from the same package. The following formula was then used for calculating the VNTR copy number: median from 10 repeat unit k-mers / max (median from 29 VNTR 5’ flank k-mers; median from 29 VNTR 3’ flank k-mers).

For direct detection of specific *TRIB3* 33-bp repeat alleles from 150 bp WGS reads, reads mapping to ±10 kb of the *TRIB3* 33-bp repeat were extracted for further analysis by text matching. Alleles with 1 to 4 copies of the 33-bp repeat were detected by searching for reads fully spanning the repeat region and additionally covering 6 bp of the 5’ and 3’ flanking regions, with a maximum allowed edit distance of 2 bp compared to the expected sequence. As the 5x33bp allele cannot be spanned by a 150 bp read, reads with partial coverage of 5 copies of the 33-bp repeat element were accepted, requiring a minimum repeat element fragment of 6 bp and a maximum edit distance of 2 bp. For each sample, alleles with <2 detection counts were excluded. Samples showing more than two alleles were excluded. To calculate multi-allelic *F*_ST_, the approach in [40] was followed.

### RNA-Seq and SNP genotype data and processing

RNA-Seq data for all tissues and all samples in GTEx v8, pre-processed by GTEx, was downloaded from the project home page [45]. All RNA-Seq samples annotated by GTEx as passing quality control were kept, and all tissues with ≥5 donors having RNA-Seq and WGS data available were kept. No further exclusion of outlier samples was applied in the calculations. Pre-processed RNA-Seq data for blood cells was also obtained for participants of the Estonian Biobank [44]. The depth-normalized RNA-Seq results (TPM for GTEx; FPKM for Estonian Biobank) were log-transformed, and Pearson’s product-moment correlation (coefficient *r*) was used to assess correlation between RNA expression level and 33-bp repeat copy number. False discovery rate was used for the correction of p values for multiple testing.

For SNP genotypes of GTEx donors, the genotyping calls from the GTEx project were used. Association between SNP genotype and RNA expression level or 33-bp repeat copy number was calculated by linear regression model for the SNP allele effect. For the calculation of linkage disequilibrium, the R package LDheatmap was used (version 0.99–7) [68].

### Luciferase reporter plasmid generation

To prepare reporter plasmids which contain approximately 0.7 kb of the human *TRIB3* 5’-flanking region (GRCh38 human reference genome chr 20 positions 380,298 to 380,987) with different copy numbers of the 33-bp repeat, the following two-step cloning procedure was carried out. Human DNA samples (received from EGCUT) were amplified by PCR using primers 5’-CCACTTCCGCTGCGAGTCTCGTG-3’ and 5’-CTCGGTACCTGAAGCTTCTGAACCACTTGC-3’, and the PCR products were cut with restriction enzymes *Acc65*I/*Mlu*I and subsequently inserted into *Acc65*I/*Mlu*I sites of the reporter vector pGL3-Basic (Promega). The inserts were analyzed for 33-bp repeat alleles and other genetic variants by Sanger sequencing and used as template for the PCR amplification with primers 5’-GCGGTTCCATCTTCCAGCGGATAG-3’ and 5’-CTCGGTACCCCGACAACAGTCACTGTTTG-3’, and the PCR products were cut with restriction enzymes *Acc65*I/*Bgl*II and inserted into *Acc65*I/*Bgl*II sites of the plasmid pGL3-Basic.

The luciferase reporter constructs TRIB3-2x33-pGL3 and TRIB3-5x33-pGL3 contain two or five copies of the 33-bp repeat, respectively. All other nucleotides of their inserts are identical: both constructs differ from the GRCh38 reference genome at a number of positions, all of which correspond to common SNPs (genotypes in the plasmids: rs575062197:delA, rs2092474:C, rs7267577:A, rs6115787:A, rs7261666:G, and rs7263534:C).

To prepare the series of luciferase reporter plasmids TRIB3(ref)-(1/2/3/4/5)x33-pGL3, carrying 1 to 5 copies of the 33-bp repeat with flanking sequences identical to the GRCh38 human reference genome (i.e., all SNPs in the region are represented by the reference allele), a genomic DNA fragment containing 3x33bp copies and matching exactly to the human reference genome chr 20 region 380,298–380,987 was used as a 33-bp repeat flanking sequence donor for fragments carrying 1, 2, 4 or 5 copies of 33-bp repeat.

Sanger sequencing was used to confirm the nucleotide sequences of all plasmids.

### Dual-luciferase reporter assay

HepG2 and H1299 cells grown in 96-well plates were cotransfected in triplicate wells with 40 ng of firefly luciferase-encoding plasmid (either pGL3-Basic or reporter constructs containing the *TRIB3* promoter region; described above) and 6 ng of *Renilla* luciferase-encoding plasmid (pRL-TK; Promega) using polyethylenimine (PEI-MAX 40,000; Polysciences Inc. #24765). Where indicated, 2.5 ng of the expression plasmid encoding human ATF4 (ATF4-pCG) [30], or the corresponding empty vector (pCG), was included in the transfection mixture. Before each transfection, fresh dilutions of reporter construct stocks were prepared, and DNA concentration was verified using NanoDrop 1000 (Thermo Scientific).

HepG2 cells were left to recover for 20 h after transfection and were subsequently incubated for 10 h in treatment medium (glucose-free DMEM (without sodium pyruvate; Gibco) supplemented with 10% dialyzed FCS (Sigma-Aldrich) and D-glucose at a final concentration of 4.5 g/l). Tunicamycin (2 μg/ml) and sodium arsenite (20 μM) (both obtained from Sigma-Aldrich) were added where indicated. To induce glucose deprivation, cells were washed twice with PBS and incubated in glucose-free treatment medium. H1299 cells were left to recover for 20 h after transfection and were subsequently either mock-treated or treated with 30 μM sodium arsenite in the cell growth medium (DMEM, without sodium pyruvate (Gibco) supplemented with 10% FCS (PAA)) for 10 h.

Firefly and *Renilla* luciferase activities were measured using a dual-luciferase assay (Promega) as described previously [3, 30]. The firefly luciferase activity was normalized to the *Renilla* luciferase activity, and the activities of the reporter constructs containing *TRIB3* promoter region are presented relative to the results from the promoterless pGL3-Basic plasmid in the same experimental conditions.

## Supporting information

S1 Fig PCR analysis of the *TRIB3* 33-bp repeat copy number in Estonian Biobank participants with the highest estimated repeat copy number based on the k-mer counting technique. The k-mer quantification result is shown below the gel. The PCR was performed as in Fig 1B and 1D, and the products generated from HepG2 cell line DNA are shown as a control. Lane M designates a 100 bp DNA ladder molecular weight marker.
(TIF)

S1 Table Correlation of TRIB3 33-bp repeat copy number and TRIB3 gene expression in GTEx tissues. All tissues with ≥5 samples of RNA-Seq and whole genome sequencing data from the same donor were included. The k-mer counting method was used to estimate the TRIB3 33-bp repeat copy number from genome sequencing reads, and TRIB3 gene expression was obtained from RNA-Seq data. Correlation was calculated using Pearson's product-moment correlation method. False discovery rate was used to correct the p values.
(DOCX)

S1 Dataset Numerical data. (XLSX)
