# Supplementary figures and images for: A human-specific VNTR in the TRIB3 promoter causes gene expression variation between individuals
Source: PLoS Genet. 2020 Aug 3;16(8):e1008981. doi: 10.1371/journal.pgen.1008981 (PMC7425993; doi:10.1371/journal.pgen.1008981)

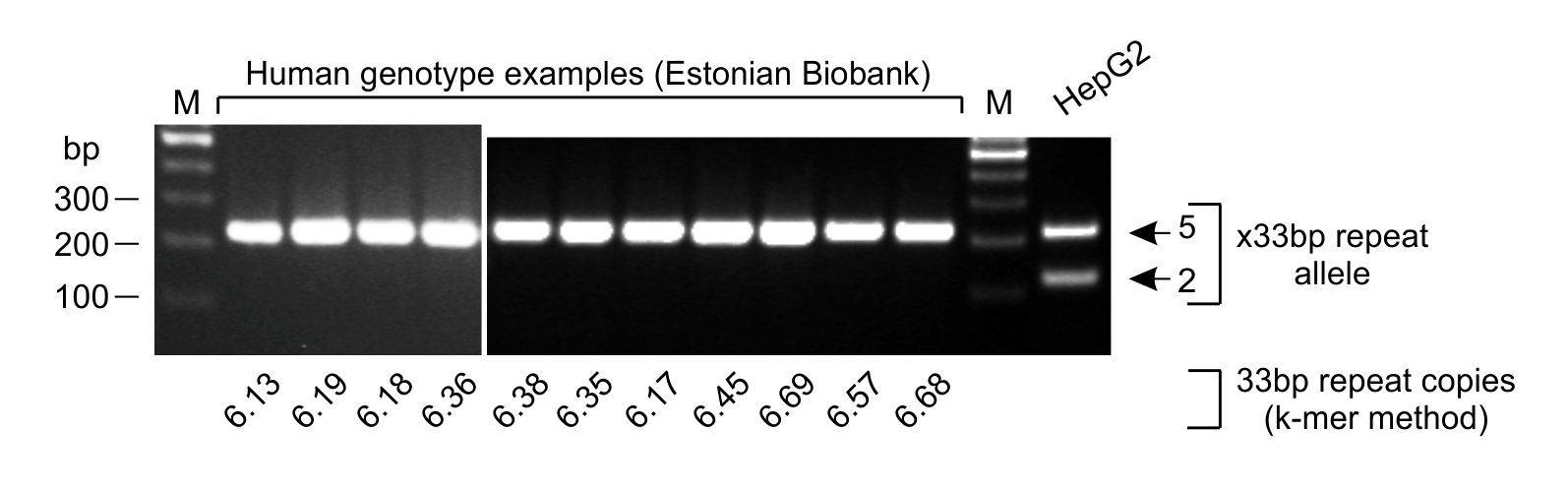

Supplement: S1 Fig — The k-mer quantification result is shown below the gel. The PCR was performed as in Fig 1B and 1D, and the products generated from HepG2 cell line DNA are shown as a control. Lane M designates a 100 bp DNA ladder molecular weight marker. (TIF) [file pgen.1008981.s001.tif]
