# Supplementary material for: A human-specific VNTR in the TRIB3 promoter causes gene expression variation between individuals
Source: PLoS Genet. 2020 Aug 3;16(8):e1008981. doi: 10.1371/journal.pgen.1008981 (PMC7425993; doi:10.1371/journal.pgen.1008981)
Supplement: S1 Table — All tissues with ≥5 samples of RNA-Seq and whole genome sequencing data from the same donor were included. The k-mer counting method was used to estimate the TRIB3 33-bp repeat copy number from genome sequencing reads, and TRIB3 gene expression was obtained from RNA-Seq data. Correlation was calculated using Pearson's product-moment correlation method. False discovery rate was used to correct the p values. (DOCX) [file pgen.1008981.s002.docx]

**Supplementary Table S1**

Correlation of TRIB3 33-bp repeat copy number (k-mer method) and TRIB3 gene expression (RNA-Seq) in GTEx tissues (Pearson's product-moment correlation).

| Tissue | Correlation p value (unadjusted) | Correlation p value (FDR-adjusted) | Correlation coefficient | Sample size |
| --- | --- | --- | --- | --- |
| Adipose - Subcutaneous | 2.58E-06 | 1.24E-05 | 0.226 | 426 |
| Adipose - Visceral (Omentum) | 9.78E-05 | 3.70E-04 | 0.212 | 332 |
| Adrenal Gland | 7.25E-03 | 1.28E-02 | 0.193 | 193 |
| Artery - Aorta | 9.97E-16 | 1.76E-14 | 0.448 | 290 |
| Artery - Coronary | 5.44E-03 | 1.03E-02 | 0.212 | 171 |
| Artery - Tibial | 2.63E-17 | 1.39E-15 | 0.393 | 429 |
| Bladder | 9.76E-01 | 9.87E-01 | -0.008 | 18 |
| Brain - Amygdala | 6.16E-03 | 1.13E-02 | 0.270 | 102 |
| Brain - Anterior cingulate cortex (BA24) | 9.33E-03 | 1.54E-02 | 0.235 | 121 |
| Brain - Caudate (basal ganglia) | 2.97E-04 | 8.74E-04 | 0.280 | 163 |
| Brain - Cerebellar Hemisphere | 2.43E-01 | 2.80E-01 | 0.097 | 146 |
| Brain - Cerebellum | 1.50E-02 | 2.34E-02 | 0.190 | 164 |
| Brain - Cortex | 3.14E-04 | 8.75E-04 | 0.286 | 155 |
| Brain - Frontal Cortex (BA9) | 9.57E-03 | 1.54E-02 | 0.208 | 154 |
| Brain - Hippocampus | 9.64E-04 | 2.43E-03 | 0.275 | 141 |
| Brain - Hypothalamus | 4.42E-02 | 6.01E-02 | 0.167 | 146 |
| Brain - Nucleus accumbens (basal ganglia) | 7.81E-08 | 5.17E-07 | 0.406 | 163 |
| Brain - Putamen (basal ganglia) | 4.43E-07 | 2.35E-06 | 0.414 | 138 |
| Brain - Spinal cord (cervical c-1) | 1.86E-03 | 4.10E-03 | 0.308 | 100 |
| Brain - Substantia nigra | 3.39E-05 | 1.38E-04 | 0.420 | 91 |
| Breast - Mammary Tissue | 1.70E-02 | 2.50E-02 | 0.144 | 274 |
| Cells - Cultured fibroblasts | 9.51E-02 | 1.20E-01 | 0.081 | 424 |
| Cells - EBV-transformed lymphocytes | 2.19E-01 | 2.58E-01 | 0.108 | 131 |
| Cervix - Ectocervix | 9.82E-02 | 1.21E-01 | 0.585 | 9 |
| Cervix - Endocervix | 1.94E-03 | 4.12E-03 | 0.848 | 10 |
| Colon - Sigmoid | 2.31E-08 | 1.75E-07 | 0.363 | 223 |
| Colon - Transverse | 1.22E-03 | 2.95E-03 | 0.197 | 267 |
| Esophagus - Gastroesophageal Junction | 1.68E-10 | 2.23E-09 | 0.409 | 226 |
| Esophagus - Mucosa | 5.78E-01 | 6.12E-01 | 0.029 | 379 |
| Esophagus - Muscularis | 8.53E-17 | 2.26E-15 | 0.422 | 355 |
| Fallopian Tube | 2.89E-01 | 3.26E-01 | 0.398 | 9 |
| Heart - Atrial Appendage | 4.24E-04 | 1.12E-03 | 0.206 | 288 |
| Heart - Left Ventricle | 5.00E-02 | 6.63E-02 | 0.114 | 294 |
| Kidney - Cortex | 4.97E-01 | 5.38E-01 | 0.098 | 50 |
| Liver | 2.03E-01 | 2.45E-01 | 0.103 | 155 |
| Lung | 4.23E-02 | 5.90E-02 | 0.101 | 407 |
| Minor Salivary Gland | 9.87E-01 | 9.87E-01 | -0.002 | 92 |
| Muscle - Skeletal | 1.26E-08 | 1.11E-07 | 0.246 | 520 |
| Nerve - Tibial | 5.83E-09 | 6.18E-08 | 0.290 | 388 |
| Ovary | 5.25E-06 | 2.32E-05 | 0.386 | 131 |
| Pancreas | 8.57E-03 | 1.47E-02 | 0.171 | 234 |
| Pituitary | 4.24E-07 | 2.35E-06 | 0.380 | 167 |
| Prostate | 1.50E-03 | 3.46E-03 | 0.255 | 152 |
| Skin - Not Sun Exposed (Suprapubic) | 3.44E-01 | 3.79E-01 | -0.050 | 362 |
| Skin - Sun Exposed (Lower leg) | 7.47E-01 | 7.76E-01 | 0.015 | 444 |
| Small Intestine - Terminal Ileum | 6.52E-02 | 8.43E-02 | 0.164 | 127 |
| Spleen | 3.31E-03 | 6.67E-03 | 0.229 | 163 |
| Stomach | 1.51E-04 | 5.35E-04 | 0.240 | 245 |
| Testis | 3.40E-03 | 6.67E-03 | 0.191 | 233 |
| Thyroid | 3.59E-02 | 5.14E-02 | 0.101 | 435 |
| Uterus | 2.44E-04 | 8.09E-04 | 0.349 | 106 |
| Vagina | 1.58E-02 | 2.39E-02 | 0.228 | 112 |
| Whole Blood | 2.82E-04 | 8.74E-04 | 0.165 | 483 |
